# Etorphine-Ketamine Constant Rate Infusion for Maintenance of Anaesthesia in a Compromised White Rhinoceros (*Ceratotherium simum*)

**DOI:** 10.1155/2019/4309043

**Published:** 2019-03-12

**Authors:** Friederike Pohlin, Peter Buss, Michele Miller, Gerhard Steenkamp, Robin Gleed, Luke Poore, Jordyn Boesch, Gareth Zeiler

**Affiliations:** ^1^Department of Paraclinical Sciences, Centre for Veterinary Wildlife Studies, Faculty of Veterinary Science, University of Pretoria, Private Bag X04, Onderstepoort 0110, South Africa; ^2^Veterinary Wildlife Services, South African National Parks, Kruger National Park, Private Bag X402, Skukuza 1350, South Africa; ^3^South African National Research Foundation, DST/NRF Centre of Excellence for Biomedical Tuberculosis Research, South African Medical Research Council Centre for Tuberculosis Research, Division of Molecular Biology and Human Genetics, Faculty of Medicine and Health Science, Stellenbosch University, Cape Town 8000, South Africa; ^4^Department of Companion Animal Clinical Studies, Faculty of Veterinary Science, University of Pretoria, Private Bag X04, Onderstepoort 0110, South Africa; ^5^Section of Anaesthesiology, Department of Clinical Sciences, Cornell University College of Veterinary Medicine, Ithaca, NY 14853, USA

## Abstract

A subadult white rhinoceros bull presented for oesophageal endoscopic evaluation and foreign body removal under general anaesthesia. The animal had a history of nasal and oral regurgitation of water and ingesta with weight-loss for 6 days prior to the procedure and had been diagnosed with oesophageal obstruction caused by a bailing wire. Anaesthesia was induced with intramuscular etorphine and azaperone delivered remotely by dart, followed by an intravenous bolus of ketamine. The trachea was intubated, and anaesthesia was maintained with an etorphine-ketamine constant rate infusion (CRI). The rhinoceros did not respond predictably to induction of anaesthesia and developed life-threatening systemic hypotension throughout the 90-minute procedure. A mega-vertebrate demand ventilator was successfully used to provide intermittent positive pressure ventilation when the rhinoceros developed apnoea. This case report describes the maintenance of anaesthesia of a white rhinoceros using an etorphine-ketamine CRI and the causes and management of hypotension and respiratory impairment observed in this patient.

## 1. Introduction

The Southern white rhinoceros (*Ceratotherium simum*) is considered as “near-threatened” by the International Union for the Conservation of Nature (IUCN) Red List of Threatened Species [1]. As the need to perform elective and emergency diagnostic and surgical procedures has increased, techniques for safe and effective anaesthesia of rhinoceroses are required [2]. Due to their size and physiology, the anaesthetic management of rhinoceroses is challenging [3–5]. Adult, free-ranging rhinoceroses are typically immobilised using etorphine-azaperone combinations with butorphanol [2, 4, 6]. To deepen and prolong anaesthesia, supplemental administration of ketamine, opioid agonists, *α*2-adrenoceptor agonists, benzodiazepine agonists, guaifenesin, propofol, or inhalant anaesthetics (isoflurane or sevoflurane), given on their own or in combination, have been reported [7–17]. However, some of these drugs are logistically challenging to use (i.e., gas anaesthetics) and may complicate anaesthesia. This paper describes a case in which a constant rate infusion (CRI) of etorphine and ketamine was administered for maintenance of anaesthesia in a compromised wild white rhinoceros temporarily housed in an outdoor enclosure.

## 2. Case Presentation

A subadult white rhinoceros bull presented for an oesophageal endoscopic evaluation and foreign body removal under general anaesthesia. This animal had been captured in the wild and temporarily housed in a Veterinary Wildlife Service purpose build outdoor enclosure (boma) in Skukuza, Kruger National Park (23°49'60”S, 31°30'0”E), South Africa, over the previous 11 weeks. It had a six-day history of nasal and oral regurgitation of food and water immediately after eating and drinking, inappetence, and weight loss, from 1,120 kg at capture to 1,048 kg during confinement. One day after the acute onset of clinical signs, the animal was immobilised using 2.4 *μ*g/kg etorphine (2.5 mg Captivon®, Wildlife Pharmaceuticals, 9.8 mg/mL) and 19 *μ*g/kg azaperone (20 mg Azaperone tartrate, Wildlife Pharmaceuticals, 50 mg/mL), followed by an intravenous bolus of butorphanol (10 times the etorphine dose in mg [Wildlife Pharmaceuticals, 50 mg/ml]). An oesophageal impaction caused by a food bolus at the entrance to the oesophagus was diagnosed. An endotracheal tube was placed into the trachea of the animal, the cuff inflated, and the oesophagus lavaged with water to resolve the impaction. Ten litres of water were administered per rectum to treat hypovolaemia, and the animal received systemic antibiotics (10 mg/kg enrofloxacin i.m. [Baytil®, Bayer, 100mg/mL] and 30 mg/kg florfenicol i.m. [Nuflor®, MSD, 300mg/mL]) and a non-steroidal anti-inflammatory drug (0.6 mg/kg Meloxicam i.v. [Metacam®, Boehringer Ingelheim, 20mg/mL]). Chemical immobilisation was reversed after 75 minutes by administering naltrexone inravenously (Trexonil®, Wildlife Pharmaceuticals, 50 mg/mL) at 20 times the etorphine-dose in mg.

Clinical signs did not resolve and the rhinoceros was re-immobilised two days later using the same protocol. A food bolus was removed manually from the caudal pharynx and a rigid linear foreign body, thought to be bailing wire, was palpated at the entrance to the oesophagus. Attempts to remove the wire manually failed and the rhinoceros rapidly decompensated, demonstrating frequent apnoeic periods of increasing duration. No further attempts were made in fear of further compromising the safety of the rhinoceros and chemical immobilisation was reversed after 55 minutes by administering 430 mg of naltrexone intravenously.

A multidisciplinary team of veterinarians was assembled two days later in order to perform endoscopic evaluation and surgical removal of the wire under general anaesthesia.

On presentation, the rhinoceros showed bilateral nasal discharge containing ingesta and appeared depressed. The body condition score was poor with pronounced skin folds on the ventral thoracic wall and abdomen consistent with weight loss.

Anaesthesia was induced using a combination of 2.5 mg etorphine and 40 mg azaperone administered intramuscularly in the left nuchal hump, via a CO_2_-propelled 3-mL dart fitted with a 2.0 x 60-mm plain needle (Daninject ApS, Børkop, 33805, Denmark). At 25 minutes after darting, the standing rhinoceros could be safely approached and blindfolded. A 100 mg intravenous bolus of ketamine (Ketamine Fresenius 10%, Bodene Intramed, 100 mg/ml) was hand-injected into an auricular vein and the rhinoceros became sternally recumbent within two minutes. The rhinoceros was positioned in left lateral recumbency, 12.5 mg butorphanol were administered intravenously, and a cold-water enema was started. A 12-gauge catheter (Jelco, Smiths Medical Ltd, South Africa) was placed into the left cephalic vein and a constant flow of lactated Ringer's solution (Intramed Ringer's-Lactate solution, Fresenius Kabi; 5 mL/kg/hour) was provided. A 16-gauge catheter (Jelco, Smiths Medical Ltd, South Africa) was placed into a vein of the left ear and a CRI of etorphine and ketamine was administered at 60 mL/hour (2 mg etorphine and 400 mg ketamine diluted in 1,000 mL lactate Ringer's solution with a final dilution of 2 *μ*g/mL and 0.4 mg/mL etorphine and ketamine, respectively). The initial flow rate resulted in a dose rate of 0.12 *μ*g/kg/hour and 24 *μ*g/kg/hour for etorphine and ketamine, respectively.

To ensure an open and secured airway during the procedure, orotracheal intubation was performed by manual palpation using a cuffed 22-mm silicon endotracheal tube (ET22, Surgivet, Smith Medical Pm Inc., USA). Oxygen was delivered at a constant rate of 30 L/min through the endotracheal tube.

Anaesthesia was monitored by evaluation of respiratory rate (RR) through observation of thoracic excursions, and heart rate (HR) by auscultation.

A multiparameter anaesthetic monitor (CardioCap/5, Datex-Ohmeda, GE Healthcare) was used to monitor end-tidal carbon dioxide partial pressure (PE'CO_2_) and the electrical activity of the heart. Electrocardiography (ECG) pads were placed in a standard base-apex configuration. A 20-gauge catheter (Jelco, Smiths Medical Ltd, South Africa) was placed in an auricular artery of the right ear and connected to an electronic strain gauge transducer and the anaesthetic monitor to measure systolic-, mean- and diastolic arterial blood pressure (BP). The transducer was zeroed to atmospheric pressure at the level of the* manubrium sterni*.

**Figure 1:**
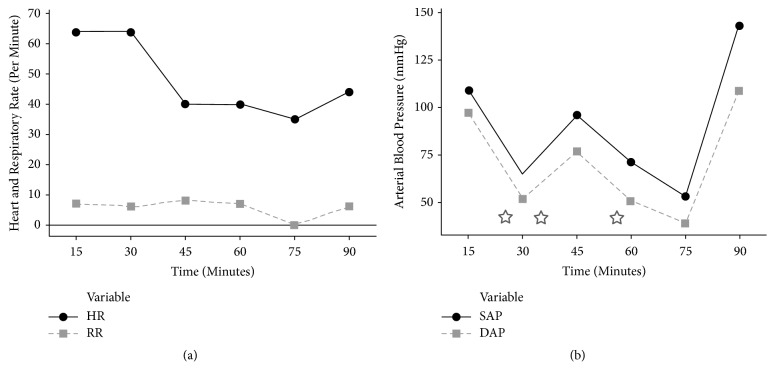
(a) Course of the respiratory rate (RR) (breaths per minute) and heart rate (HR) (beats per minute); (b) diastolic arterial blood pressure (DAP) (mmHg), and systolic arterial blood pressure (SAP) (mmHg) during the anaesthetic procedure in a subadult white rhinoceros. Time is the elapsed time after recumbency. Medetomidine administration (stars) caused an increase in blood pressure resulting from peripheral vasoconstriction. IPPV was initiated at minute 75.

Data were recorded at 5-minute intervals during the anaesthetic period. The values for HR, RR, systolic BP (SAP), diastolic BP (DAP), and interventions during the procedure are summarised in Figure 1.

During the first hour of anaesthesia, the rhinoceros breathed spontaneously at a rate of 7 (mean ± SD 0.75; range 6-8) breaths per minute (brpm) and had PE'CO_2_ values of 52 (mean ± SD 3.65; range 48-60) mmHg. At 25 minutes after positioning in lateral recumbency, the rhinoceros developed severe hypotension: SAP decreased to ≤ 60 mmHg and pulse pressure decreased to ≤ 10 mmHg. One milligram of medetomidine (1 *μ*g/kg, Domitor®, Zoetis, 1 mg/mL) was administered intravenously and the infusion rate of the etorphine-ketamine CRI reduced to one-third. Within one minute, the rhinoceros reacted with an increase in SAP (from 55 to 119 mmHg), DAP (from 52 to 97 mmHg), and pulse pressure (from 3 to 22 mmHg). HR decreased from 64 beats per minute (bepm) to 40 bepm. The same procedure was repeated successfully 10 (minute 35) and 30 (minute 55) minutes later, as hypotension reoccurred. HR, however, remained stable at 40 bepm. After the third medetomidine bolus, BP continued decreasing and the rhinoceros developed apnoea. Intermittent positive pressure ventilation (IPPV, inspiratory pressure ~30 cm H_2_O, RR 10 brpm) was initiated (at minute 75) using a portable mega-vertebrate demand ventilator powered by compressed oxygen, delivering a fraction of inspired oxygen of approximately 0.42 (www.incaseofanesthesia.com). Within two minutes of initiating IPPV, BP values returned to normal and the rhinoceros continued breathing spontaneously at a rate of 4 brpm.

The endoscopic examination revealed rupture of the cervical oesophagus accompanied by extensive tissue destruction and debris with drainage of saliva and food material into the fascial planes of the neck. Due to a grave prognosis, the anaesthetised rhinoceros was euthanised at 90 minutes after induction by administering 80 mL of a saturated solution of succinylcholine chloride (Suxamethonium chloride, Kyron Laboratories) and potassium chloride (Kyron Laboratories, 1 g powder) intravenously.

## 3. Discussion

Our case report describes intravenous maintenance of anaesthesia using an etorphine-ketamine CRI in a compromised white rhinoceros suffering from an oesophageal obstruction.

Oesophageal obstruction (choke) is frequently described in the domestic horse, a species related to the rhinoceros [18]. A case of a regurgitating southern black rhinoceros (*Diceros bicornis minor*) undergoing endoscopic examination of the oesophagus has been described by Radcliffe et al. (1998) [19]. That particular animal was immobilised with a combination of etorphine and azaperone and maintained with isoflurane. We used the same standard drug combination to immobilise our rhinoceros, but maintained anaesthesia with an etorphine-ketamine CRI.

We opted to use the standard etorphine-azaperone combination, as it was familiar to the wildlife veterinarian—a practice that improves overall safety [20, 21]. Etorphine, a potent opioid, is the most frequently used agent for chemical immobilisation of white rhinoceroses and results in hypertension and tachycardia [2, 4]. The inclusion of azaperone with etorphine in the immobilising drug combination reduces the opioid-induced hypertension and shortens the induction time [22].

Our animal responded with a prolonged induction time compared to its previous immobilisations, where it had attained recumbency within ten minutes. Dehydration and electrolyte and acid-base imbalances associated with regurgitation may have prolonged the induction time by altering drug uptake and distribution [18, 23]. As reported by Atkinson et al. (2002) in a greater one-horned Indian rhinoceros (*Rhinoceros unicornis*), the history of repeated etorphine-based immobilisations might have led to an opioid drug tolerance in our rhinoceros [8]. Additionally, residual activity of the opioid-antagonist naltrexone from the latest immobilisation may also have prevented complete immobilisation of the rhinoceros by interfering with the immobilising drugs due to its long half-life [4].

An intravenous bolus of ketamine was ultimately administered to complete induction of anaesthesia, followed by an intravenous bolus of butorphanol after recumbency was achieved. Intravenous ketamine has been used as a co-induction agent in combination with guaifenesin or diazepam in premedicated white rhinoceroses that were also compromised [10, 15]. Butorphanol has commonly been used, and often preferred, as a supplemental agent in etorphine-based anaesthesia protocols, as it modulates opioid receptor effects and thereby reduces respiratory depression [22, 24, 25]. Moreover, butorphanol alone, and in combination with other tranquilizers, has produced good results for standing and recumbent chemical restraint of compromised white rhinoceroses and an intravenous butorphanol CRI has been used to maintain standing sedation for a minor surgical procedure in a black rhinoceros [25, 26]. However, in the present case, with a history of multiple etorphine-based immobilisations, it was reasoned that butorphanol, alone or in combination, would not provide an adequate state of immobilisation.

We maintained anaesthesia using an etorphine-ketamine CRI. In clinical settings, inhalation drugs such as halothane [16], isoflurane [10, 15, 19, 27], and sevoflurane [14] have been used to maintain anaesthesia in rhinoceroses. However, inhalant anaesthesia requires bulky equipment, especially for large animals, and may be impractical and expensive to use outside of a clinical setting [28]. Thus, most authors have used intermittent intravenous boluses of ketamine [8, 9, 17, 29], ketamine-detomidine-guaifenesin [12], detomidine [29], etorphine-detomidine [13], etorphine-azaperone [16], or propofol [29] to maintain anaesthesia in rhinoceroses. The depth of anaesthesia produced by injectable anaesthetics is proportional to the plasma concentration of the given drug. Administering intermittent intravenous boluses results in peaks and troughs of the plasma drug concentration and, hence, fluctuations of clinical and undesirable physiological effects. These undesirable physiological effects can be minimised by administering a drug as a CRI where a near-constant plasma concentration within the therapeutic range can be achieved, producing a more consistent desired clinical effect [30]. In rhinoceroses, CRIs of 5% guaifenesin-ketamine [31] and medetomidine-ketamine [10] have been described to maintain anaesthesia, or supplement isoflurane-anaesthesia. The use of an etorphine-ketamine CRI has not yet been described in rhinoceroses and, thus, undesirable physiological effects of this drug-combination remain to be investigated in healthy animals. However, etorphine-ketamine- and medetomidine CRI has been used in healthy wild impala (*Aepyceros melampus*); BP, RR, and HR remained within physiological reference ranges, but animals experienced hypoxaemia [32].

The administration of the etorphine-ketamine CRI in our compromised rhinoceros resulted in surgical plane of anaesthesia with hypotension as the main complication encountered.

Rhinoceroses receiving etorphine are known to experience systemic hypertension, hypoxaemia, and acidosis [3–5]—hypotension represents a rather unusual complication. Even though oxygen was provided, our patient was suspected to be suffering from etorphine-induced hypoxaemia and acidosis, which likely resulted in poor cardiovascular performance and vasodilation. Additional inflammatory mediated vasodilation together with the hypovolaemic status of the dehydrated animal likely caused the profound hypotension [5, 33, 34]. Stimulation of afferent and efferent pathways in the vagus nerve, resulting from manipulation of the caudal pharyngeal region, may have contributed to the low BP [33].

Arterial BP is the product of cardiac output and systemic vascular resistance and, thus, any increase in either increases BP [33]. The choice of cardiovascular supportive drug depends on the suspected underlying pathology and the patient's response to therapy. Dobutamine and phenylephrine infusions have been used to maintain mean arterial BP in white and black rhinoceroses anaesthetised with isoflurane in oxygen [7, 10]. However, the inotropic and chronotropic effects of dobutamine lead to an increase in myocardial oxygen demand and may have been harmful in a rhinoceros that suffered from etorphine-induced hypoxaemia associated with poor myocardial oxygenation [33, 34]. The potent vasoconstrictor phenylephrine in contrast is known to impair peripheral oxygen delivery and skeletal muscle blood flow [35]. During a hypotensive crisis, it should be used cautiously. Hence, we administered a low dose of medetomidine (1 *µ*g/kg), an alpha-2 adrenergic agonist. Medetomidine causes peripheral vasoconstriction and, at low doses, it leads to a transient increase in arterial BP without necessarily affecting heart rate [36, 37]. Furthermore, it is known to decrease systemic oxygen demand by decreasing cellular metabolism, which might have been of advantage in our rhinoceros [36].

Despite our efforts, at one hour of anaesthesia, the rhinoceros developed apnoea—an initial sign of cardiopulmonary arrest [38]. Coronary blood flow and, thus, myocardial perfusion largely depend on the DAP [39]. Our rhinoceros demonstrated severe hypotension that, in combination with etorphine-induced hypoxaemia, most likely led to poor oxygen-supply of the myocardium and impaired myocardial performance.

IPPV was initiated immediately after the onset of apnoea. We used the portable mega-vertebrate demand ventilator for IPPV, which has been shown to significantly improve oxygenation in etorphine-immobilised white rhinoceroses in zoo [40]. To the authors' knowledge, this is the first report of the mega-vertebrate demand ventilator being used for IPPV of an anaesthetised wild white rhinoceros temporarily housed in a boma. Within two minutes of initiating IPPV, DAP and SAP increased dramatically, most likely because of improved myocardial oxygenation and stimulation of cardiac activity [33]. It might have been more beneficial to start IPPV earlier during anaesthesia to maintain oxygenation from the beginning and decrease the risk for hypoxia-induced hypotension and myocardial insufficiency.

## 4. Conclusion

This report provides useful information on a novel anaesthetic protocol used in a compromised white rhinoceros receiving special cardiovascular and respiratory support. We believe that the use of an etorphine-ketamine CRI is an option for intravenous maintenance of anaesthesia in rhinoceroses. Further investigation of its use in healthy rhinoceroses is warranted to draw any conclusions on expected physiological side effects. Attention should be given to maintain oxygenation and tissue perfusion by administering a partial opioid agonist-antagonist and by providing oxygen insufflation and fluid resuscitation. The importance of having vascular access, tracheal intubation, and IPPV devices at hand when working with compromised white rhinoceroses cannot be overstated.
